# Laboratory evaluation of four HIV/syphilis rapid diagnostic tests

**DOI:** 10.1186/s12879-018-3567-x

**Published:** 2019-01-03

**Authors:** Annelies Van Den Heuvel, Hilde Smet, Irena Prat, Anita Sands, Willy Urassa, Katrien Fransen, Tania Crucitti

**Affiliations:** 1 HIV/STI Reference Laboratory, Department of Clinical Sciences, Institute of Tropical Medicine, Antwerp, Belgium; 2 World Health Organisation (WHO), Geneva, Switzerland

## Abstract

**Background:**

Sexually transmitted infections, such as HIV and syphilis, are one of the major health care problems worldwide, especially in low- and middle income countries. HIV screening programmes have been widely used for many years. The introduction of rapid point-of-care tests (RDTs) that can detect both HIV and syphilis, using one single blood specimen, would be a promising tool to integrate the detection of syphilis into HIV programmes and so improve the accessibility of syphilis testing and treatment.

**Methods:**

As part of the World Health Organization pre-qualification of in vitro diagnostics assessment, the laboratory performance of four dual HIV-Syphilis rapid diagnostic tests (SD Bioline HIV/Syphilis Duo, DPP HIV-Syphilis Assay, Multiplo Rapid TP/HIV Antibody Test and Insti Multiplex HIV-1/HIV-2/Syphilis Antibody Test) was assessed using a well characterized multiregional panel of stored sera specimens.

**Results:**

In total 400 specimens were tested with each assay, resulting in excellent sensitivities and specificities for HIV, ranging from 99.5 to 100% and from 93.5 to 99.5%, respectively. Results obtained for the *Treponema pallidum* antibodies were lower, with the lowest sensitivity of 73.5% for Multiplo and the highest of 87% for SD Bioline. Specificities ranged from 99.0 to 100%.

**Conclusion:**

Although these results suggest that the tests could further improve in accuracy in detection of treponemal antibodies, their introduction into screening programmes to increase the accessibility of HIV/Syphilis diagnosis and treatment for difficult to reach populations in the world is promising.

## Background

Sexually transmitted infections (STIs) are one of the major health care problems worldwide, especially in resource-poor settings. HIV infects approximately 1.8 million people a year with 36.7 million people living with HIV at the end of 2016; and it is estimated that on a yearly basis there are 5.6 million new syphilis infections (http://www.who.int/mediacentre/factsheets/fs360/en/). In 2012, more than 900,000 pregnant women were infected with syphilis, which resulted in approximately 35,000 adverse birth outcomes (http://www.who.int/mediacentre/factsheets/fs110/en/ and http://www.who.int/reproductivehealth/topics/rtis/syphilis/pregnancy/en/). Untreated syphilis can result in serious adverse outcomes for pregnancy and can increase the risk of mother-to-child transmission of HIV [1]. As both HIV and syphilis are transmittable sexually and from mother to child, it is not surprising that co-infections are common [2].

Like most STIs, HIV and syphilis are often asymptomatic, which makes sensitive diagnostic testing particularly crucial for early detection and diagnosis, and for guidance of treatment and prevention of onward transmission. Diagnostic tests are available but often not accessible for populations living in the areas where they are needed the most, highlighting the importance of accessibility to simple and affordable tests, such as rapid diagnostic tests (RDTs). These are tests that can be used for testing at point of care, with no requirement for sophisticated laboratory equipment, or specific storage conditions; making them useful in all kinds of healthcare facilities. Since results are mostly available within 30 min, an accelerated linkage to treatment and care can be achieved. For many years, HIV screening programmes that use a validated testing algorithm of anti-HIV RDTs, have been widely used in low- and middle-income countries. Combining detection of anti-HIV and anti-treponemal antibodies in one dual RDT can integrate the detection of syphilis into HIV programmes and so improve the accessibility of syphilis testing and treatment.

Several studies have reported varied performance of currently available combined HIV/syphilis RDTs [5–24]. In this study, the laboratory-based performance of four dual HIV/Syphilis RDTs (SD Bioline HIV/Syphilis Duo (Standard Diagnostics, Republic of Korea), DPP HIV-Syphilis Assay (Chembio Diagnostics Systems, United States), Multiplo Rapid TP/HIV Antibody Test (Medmira Inc., Canada) and Insti Multiplex HIV-1/HIV-2/Syphilis Antibody Test (bioLytical Laboratories, Canada) was evaluated in comparison with a standard reference testing algorithm for HIV and syphilis, using a multiregional panel of sera.

## Methods

### Assays

As part of the World Health Organization (WHO) pre-qualification of in vitro diagnostics assessment, four dual HIV/Syphilis RDTs were evaluated between 2014 and 2016 by WHO at the Institute of Tropical Medicine (Antwerp, Belgium). SD Bioline HIV/Syphilis Duo (Standard Diagnostics, Republic of Korea, product number 06FK30; version instructions for use (IFU) 2013/05), DPP HIV-Syphilis Assay (Chembio Diagnostic Systems, United States, product number 659525; version IFU 10–6307-0Rev1) and Multiplo Rapid TP/HIV Antibody Test (Medmira, Canada, product number 815311005145; version IFU MPSIPYZIS0002EN Rev3/1) were evaluated simultaneously. The evaluation of Insti Multiplex HIV-1/HIV-2/Syphilis Antibody Test (bioLytical Laboratories, Canada, product number 90–1032; version IFU 50-1143E) was performed at a later time point. The SD Bioline and the DPP assay are lateral flow (immunochromatographic) RDTs while Multiplo and INSTI are RDTs based on the flow through (immunofiltration) principle. All assays were performed by one operator (blinded to the reference results) according to the manufacturer’s instructions for use (IFU). The test characteristics of the assays are described in Table 1.

**Table 1:** Test characteristics

	SD Bioline HIV/Syphilis Duo	DPP HIV-Syphilis Assay	Multiplo Rapid TP/HIV Antibody Test	Insti Multiplex HIV-1/HIV-2/Syphilis Antibody Test
Type of assay	Lateral flow immunochromatographic assay	Lateral flow immunochromatographic assay (dual path platform)	Vertical flow immunofiltration immunoassay (flow through)	Vertical flow immunofiltration immunoassay (flow through)
Specimen	Serum, plasma, whole blood	Serum, plasma, whole blood	Serum, plasma, whole blood	Serum, plasma, whole blood
Volume required	10 μl of serum/plasma	10 μl of serum/plasma	1 drop of whole blood/serum/plasma (35-40 μl)	50 μl of whole blood/serum/plasma
	20 μl of whole blood	1 sample loop or 10 μl of whole blood		
Time to results	15–20 min	15–25 min	Once all fluid is absorbed	Immediately after adding the Clarifying Solution
			(+/− 3 min)	
				(+/− 1–2 min)
Equipment	Timer	Timer	None	None
HIV component	HIV-1/2 recombinant antigens (gp41, gp36 and HIV-1 group O)	HIV-1/2 recombinant antigens (not specified)	HIV-1/2 synthetic peptides (gp36, gp41, gp120 and HIV-1 group O)	HIV-1/2 recombinant antigens (gp41, gp36)
TP component	Recombinant antigen (17 kDa)	IgG/IgM recombinant antigen (not specified)	IgG/IgM recombinant antigens (15 kDa, 17 kDa, 47 kDa)	Recombinant fusion proteins derived from p17 and p47 domains
Steps to perform	Add the specimen.	Add the specimen to the Sample Tainer (with buffer), mix.	Add 3 drops of buffer to the device.	Add the specimen to the Sample diluent vial, mix.
	Add 3 drops of diluent.		Add the specimen.	
		Add 2 drops to the test device at Well 1.	Place the InstantGoldCap.	Add the content of this vial to the device.
		Add 4 drops of buffer to the test device at Well 2.	Add 12 drops of buffer.	Add the Colour Developer.
			Remove the InstantGoldCap.	Add the Clarifying Solution.
			Add 3 drops of buffer.	
Result interpretation	Clear	Clear	Clear	There is no marker on the devices to indicate the position of the HIV, syphilis and control dot.

### Evaluation protocol

The evaluations were conducted according to the WHO pre-qualification protocol for performance evaluation. All personnel working on the evaluations were trained in performing and/or interpreting the assays. The results, recorded on standardized data collection sheets, were visually interpreted by the performer of the assay and independently by two other readers. Data entry into the standardized excel files was checked by a second person by visually comparing a print-out of the entered data with the raw data.

All specimens were tested in singular (initial testing). Specimens from the WHO specimen reference panel with indeterminate results (very faint doubtful shadow on the test line or discrepancies between the three readers) or results discrepant from the reference result were repeated in duplicate with the same lot (repeat testing) and (if possible) with the other lot. The result that occurred the most (at least two out of three) was recorded as the final result.

Results obtained with the assays under evaluation were compared to the results of the reference testing algorithm for HIV and Syphilis (described below).

### Panels

#### WHO specimen reference panel

All four assays were evaluated using the same characterized evaluation panel of 400 serum/plasma specimens from European, African, Asian, South American and Australian origin collected from patients/study participants attending the Institute of Tropical Medicine (ITM) clinic, collaborating sexually transmitted infection (STI) clinics and blood donation centres. All specimens were assigned a unique identification code.

The panel consisted of 200 anti-HIV antibody positive specimens, of which 100 were anti-TP (*Treponema pallidum*) antibody positive and 100 were anti-TP antibody negative, and 200 anti-HIV antibody negative specimens, of which 100 were anti-TP antibody positive and 100 anti-TP antibody negative. Separate aliquots (approximate 200 μl) of each specimen were kept stored at − 20 °C. Specimens were not thawed more than twice.

The WHO specimen reference panel was tested using the following HIV reference testing algorithms: Vironostika HIV Ag/Ab (bioMérieux, France), Enzygnost Anti-HIV 1/2 Plus (Siemens Healthcare Diagnostics, Germany) or Genscreen HIV-1/2 Version 2 (Bio-Rad, France) and INNO-LIA HIV I/II Score (Fujirebio Europe, Belgium). The Innotest HIV Antigen mAb (Fujirebio Europe, Belgium) was used to further characterize specimens in the event of discrepant results between the assay under evaluation and the reference result. The treponemal (TP) antibody status was determined by testing with the Vitros Syphilis TPA Assay (Ortho Clinical Diagnostics, USA), an automated Treponemal enzyme immunoassay (EIA), and *Treponema pallidum* passive particle agglutination (TPPA) (SERODIA-TP.PA, Fujirebio, Japan). Specimens with discrepant results to the reference result were further characterized using the BD Macro-Vue™ RPR Card Tests (Becton Dickinson, USA) for the determination of non-treponemal antibodies and/or *recom*Line Treponema IgM (MIKROGEN, Diagnostik, Germany) for the detection of treponemal IgM antibodies.

#### Commercial sera panels

The ability of the assays to detect various levels of HIV antibodies was tested by using eight HIV seroconversion panels (PRB914, PRB925, PRB926, PRB930, PRB955, PRB965, PRB968 and PRB 969; SeraCare Life Science Inc., USA), one anti-HIV mixed titre performance panel (PRB205; SeraCare Life Science Inc., USA) and the WHO international biological reference preparation panel (catalogue number 02/210; NIBSC). For Treponema pallidum (TP), one seroconversion panel (PSS901–1.2; SeraCare Life Sciences Inc., USA), one anti-TP mixed titre performance panel (PSS202 (M2); SeraCare, Life Sciences Inc., USA) and the WHO international biological reference preparation panel (catalogue number 05/122; NIBSC) were tested. All panel specimens were characterized using the same set of assays as described for the WHO specimen reference panel. After characterization the specimens were divided into smaller aliquots (approximately 200 μl) and stored at − 20 °C or − 80 °C. Specimens were not thawed more than twice.

### Performance and operational characteristics/statistics

The sensitivity and specificity values with their 95% confidence intervals (95% CI) were determined in comparison with the reference results, using the exact binominal method. Additionally, the invalid rate and inter-reader variability were calculated for each assay (for the WHO specimen reference panel). The invalid rate was expressed as the number of invalid test results over the total number of tests used (percentage). For each test band the inter-reader variability was expressed as the percentage of specimens for which initial results were differently interpreted (i.e., reactive or non-reactive or indeterminate) by the independent readers.

The operational characteristics, such as ease of use, number of steps and ease of interpretation, were assessed by the lab technician who performed the tests.

Statistical analyses (two-independent-samples-t-test, logistic regression and McNemar’s test for paired proportions) were performed using R version 3.3.2.

## Results

### WHO specimen reference panel

All 400 specimens of the WHO specimen reference panel were tested by the four RDTs. The control line was present for the 400 panel specimens on SD Bioline HIV/Syphilis Duo, DPP HIV-Syphilis Assay and Insti Multiplex HIV-1/HIV-2/Syphilis Antibody Test, indicating 100% valid results. However, for the Multiplo Rapid TP/HIV Antibody Test, invalid results were initially obtained for eight specimens, mainly due to non or incomplete absorption of the sample, and of these, two remained invalid after repeat testing (final invalid rate 2/400 = 0.5%).

As shown in Table 2, the inter-reader variability was higher on the TP component in comparison to the HIV component with SD Bioline HIV/Syphilis Duo, DPP HIV-Syphilis Assay and Multiplo Rapid TP/HIV Antibody Test: 4.0, 4.5 and 4.8% for TP versus 0, 1.0 and 0.3% for HIV, respectively. In contrast, the inter-reader variability was lower for the TP dot (1.8%) compared to the HIV dot (4.5%) for the Insti Multiplex HIV-1/HIV-2/Syphilis Antibody Test.

**Table 2:** Performance characteristics

RDT	HIV antibodies					*Treponema pallidum* antibodies					Final invalid rate (%)
	Sensitivity (%) (95% CI)		Specificity (%) (95% CI)		Inter-reader variability (%)	Sensitivity (%) (95% CI)		Specificity (%) (95% CI)		Inter-reader variability (%)	
	Initial	Final	Initial	Final		Initial	Final	Initial	Final		
SD Bioline HIV/Syphilis Duo	100 (98.2–100)	100 (98.2–100)	99.5 (97.2–100)	99.5 (97.2–100)	0	86.5 (81.0–90.9)	87.0 (81.5–91.3)	99.5 (97.2–100)	99.5 (97.2–100)	4.0	0
DPP HIV-Syphilis Assay	100 (98.2–100)	100 (98.2–100)	96.0 (92.3–98.3)	97.5 (94.3–99.2)	1.0	85.0 (79.3–89.6)	86.5 (81.0–90.9)	100 (98.2–100)	100 (98.2–100)	4.5	0
Multiplo Rapid TP/HIV Antibody Test	99.5 (97.2–100)	99.5 (97.2–100)	99.5 (97.2–100)	99.5 (97.2–100)	0.3	70.0 (63.1–76.3)	73.5 (66.8–79.5)	99.0 (96.4–99.9)	99.5 (97.2–100)	4.8	0.5
Insti Multiplex HIV-1/HIV-2/Syphilis Antibody Test	99.5 (97.2–100)	99.5 (97.2–100)	88.0 (82.7–92.2)	93.5 (89.1–96.5)	4.5	78.5 (72.2–84.0)	81.0 (74.9–86.2)	99.0 (96.4–99.9)	99.0 (96.4–99.9)	1.8	0

The overall test performance characteristics per assay are summarized in Table 2. Initial sensitivity and specificity were calculated on the initial test results, and the final sensitivity and specificity on the combined results of the initial and repeat testing.

#### Anti-HIV detection component

The sensitivities obtained for HIV were 100% (95%CI: 98.2–100%) for SD Bioline, 100% (95%CI: 98.2–100%) for DPP, 99.5% (95%CI: 97.2–100%) for Multiplo and 99.5% (95%CI, 97.2–100%) for INSTI.

Specificities for SD Bioline, DPP, Multiplo and INSTI were 99.5% (95%CI: 97.2–100%), 97.5% (95%CI: 94.3% - 99.2), 99.5% (95%CI: 97.2–100%) and 93.5% (95%CI: 89.1–96.5%), respectively. For DPP and INSTI the specificity increased slightly after repeat testing of specimens with discrepant results (results in Table 2).

In general, 92.0% of the specimens (368/400) were concordant with the reference result for the HIV antibodies in the four RDTs after first testing. All discordances (*n* = 32, in one or more RDTs) were in TP positive specimens. One HIV infection was missed in the presence of TP antibodies (Australian origin) and 31 specimens (all of African origin) were falsely reactive for HIV antibodies. Out of these, four specimens were misclassified in two or more assays (see Table 3). No specimens were misclassified for HIV in all four assays. Most specimens remained discrepant from the reference result in one or more assays after re-testing (final result versus initial report) Table 3.

**Table 3:** Details of specimens discrepant from the HIV reference result, in two or more assays

WHO N°	Origin	Result for HIV antibodies								Reference Results *Treponema pallidum*					Reference Results HIV													
		INSTI Multiplex HIV-1/HIV-2/Syphilis antibody test		DPP HIV/Syphilis Assay		SD Bioline HIV/Syphilis Duo		Multiplo rapid TP/HIV Antibody test																				
										Vitros Syphilis TPA assay		TP particle agglutination (TTPA)		Reference result for TP	Vironostika HIV Ag/Ab		Genscreen HIV 1/2 v2		Innotest HIV Ag mAb		InnoLIA HIV1/2 Score							Reference result for HIV
		Initial	Final	Initial	Final	Initial	Final	Initial	Final	S/CO	Result		Result		OD/CO	Result	OD/CO	Result	OD/CO	Result	gp120	gp41	p31	p24	p17	gp105	gp36	
140,235	Australia	FN	FN	OK	OK	OK	OK	FN	FN	89.10	R	640	R	Positive	16.80	R	33,68	R	0.70	NR	1+	1+	–	±	–	–	–	HIV-1 positive
140,058	Africa	FR	FR	OK	OK	FR	FR	OK	OK	302.00	R	2560	R	Positive	0.26	NR	0,85	NR	0.41	NR	NT							Negative
140,115	Africa	FR	OK	FR	FR	OK	OK	OK	OK	57.30	R	320	R	Positive	0.29	NR	0,31	NR	0.51	NR	NT							Negative
140,134	Africa	FR	OK	FR	OK	OK	OK	OK	OK	46.80	R	1280	R	Positive	0.31	NR	0,35	NR	0.47	NR	NT							Negative

*FR* false reactive, *FN* false non-reactive, *OK* result is concordant with the reference result, *S/CO* sample signal/cut-off, *OD/CO* optical density/cut-off, *R* reactive, *NR* non-reactive, *NT* not tested

#### Anti-TP detection component

The initial sensitivities for identifying *Treponema pallidum* antibodies were 86.5% (95%CI: 81.0–90.9%) for SD Bioline, 85.0% (95%CI: 79.3–89.6%) for DPP, 70.0% (95%CI: 63.1–76.3%) for Multiplo and 78.5% (95%CI: 72.2–84.0%) for INSTI; specificities were 99.5% (95%CI: 97.2–100%), 100% (95%CI: 98.2–100%), 99.0% (95%CI: 96.4–99.9%) and 99.0% (95%CI: 96.4–99.9%), respectively. Sensitivities and specificities improved slightly after repeat testing of specimens with discrepant results (results in Table 2).

After initial testing, 83.8% (335/400) of the results were in concordance with the *Treponema pallidum* antibodies reference results in all assays.

Four anti-TP negative specimens (4/200; one HIV negative and three HIV positive; all from South American origin), were false reactive or indeterminate for *Treponema pallidum* antibodies in at least one of the RDTs. Two remained discrepant from the reference result after repeat testing.

Independent of the HIV status, 30.5% (61/200) of the TP antibody positive samples were not detected by at least one of the assays after first testing, of which 42 were discrepant from the reference result (false non-reactive or indeterminate) in two or more assays (21%). Fourteen of these were false non-reactive on all four RDTs even after repeat testing, eight on three assays and 20 on two. Most remained false non-reactive after repeat testing (see Table 4). Comparing the TPA results of the specimens that were misclassified (false negative or indeterminate) on two or more RDTs with those that were scored correctly, showed a statistical significant difference between the two groups (*p* < 0.001, mean TPA values of 36 versus 297, respectively, *p*-value obtained by two independent samples t-test).

**Table 4:** Details of specimens discrepant from the TP reference result in two or more assays

WHO N°	Origin	INSTI Multiplex HIV-1/HIV-2/Syphilis antibody test		DPP HIV/Syphilis Assay		SD Bioline HIV/Syphilis Duo		Multiplo rapid TP/HIV Antibody test		Reference Results TP							Reference result for HIV
										Vitros Syphilis TPA assay		TP particle agglutination (TTPA)		RPR	RecomLine Treponema IgM	Reference result for TP	
		Initial	Final	Initial	Final	Initial	Final	Initial	Final	S/CO	Result		Result				
Initial result not correct in all four RDTs																	
WHO3–0008	South America	FN	FN	FN	FN	FN	FN	FN	FN	11,60	R	160	R	N	NT	Positive	Negative
140,017	Africa	FN	FN	FN	FN	FN	FN	FN	FN	11,80	R	640	R	N	NT	Positive	Negative
140,020	Africa	FN	FN	FN	FN	FN	FN	FN	FN	8,50	R	320	R	N	NT	Positive	Negative
140,087	Africa	FN	FN	FN	FN	FN	FN	FN	FN	22,00	R	320	R	N	NT	Positive	Negative
140,127	Africa	FN	FN	FN	FN	FN	FN	FN	FN	20,90	R	320	R	N	NT	Positive	Negative
140,148	Europe	FN	FN	FN	FN	FN	FN	FN	FN	7,97	R	160	R	N	NT	Positive	HIV-1 positive
140,150	Europe	FN	FN	FN	FN	FN	FN	FN	FN	4,65	R	160	R	N	NT	Positive	HIV-1 positive
140,176	Europe	FN	FN	FN	FN	FN	FN	FN	FN	11,60	R	1280	R	N	NT	Positive	HIV-1 positive
140,179	United States	FN	FN	FN	FN	FN	FN	FN	FN	5,83	R	160	R	N	NT	Positive	HIV-1 positive
140,180	Europe	FN	FN	FN	FN	FN	FN	FN	FN	5,79	R	320	R	N	NT	Positive	HIV-1 positive
140,207	Europe	FN	FN	FN	FN	FN	FN	FN	FN	36,40	R	640	R	N	NT	Positive	HIV-1 positive
140,215	Africa	FN	FN	FN	FN	FN	FN	FN	FN	13,20	R	160	R	N	NT	Positive	HIV-1 positive
140,231	Asia	FN	FN	FN	FN	FN	FN	FN	FN	5,97	R	80	R	N	NT	Positive	HIV-1 positive
991,126	Africa	FN	FN	FN	FN	FN	FN	FN	FN	5,16	R	160	R	N	NT	Positive	HIV-1 positive
Initial result not correct in three RDTs and one indeterminate result																	
140,016	Africa	FN	FN	FN	FN	IND	OK	FN	FN	19,50	R	640	R	N	NT	Positive	Negative
140,037	Africa	FN	FN	FN	FN	IND	OK	FN	FN	44,40	R	640	R	R	NT	Positive	Negative
140,107	Africa	FN	FN	FN	FN	IND	IND	FN	FN	24,90	R	640	R	N	NT	Positive	Negative
140,109	Africa	FN	FN	FN	FN	IND	IND	FN	FN	18,30	R	320	R	R	NT	Positive	Negative
140,209	Europe	FN	FN	FN	OK	IND	FN	FN	FN	29,10	R	320	R	N	NT	Positive	HIV-1 positive
Initial result not correct in two RDTs and two indeterminate results																	
140,006	Africa	FN	FN	IND	IND	IND	OK	FN	FN	59,80	R	640	R	N	NT	Positive	Negative
140,170	Europe	FN	FN	IND	FN	IND	OK	FN	FN	14,40	R	160	R	N	NT	Positive	HIV-1 positive
140,208	Europe	FN	FN	IND	FN	IND	OK	FN	FN	40,90	R	640	R	N	NT	Positive	HIV-1 positive
Initial result not correct in tree RDTs																	
140,009	Africa	FN	FN	FN	OK	OK	OK	FN	FN	54,80	R	1280	R	N	NT	Positive	Negative
140,149	Europe	OK	OK	FN	FN	FN	FN	FN	FN	42,60	R	320	R	N	NT	Positive	HIV-1 positive
991,110	Africa	FN	FN	FN	FN	FN	FN	OK	OK	8,68	R	80	R	N	NT	Positive	HIV-1 positive
Initial result not correct in two RDTs and one indeterminate result																	
140,085	Africa	FN	FN	IND	FN	OK	OK	FN	FN	7,90	R	160	R	N	NT	Positive	Negative
140,091	Africa	FN	FN	OK	OK	IND	IND	FN	IND	31,50	R	640	R	N	NT	Positive	Negative
140,146	Europe	FN	FN	IND	IND	OK	OK	FN	FN	19,30	R	80	R	N	NT	Positive	HIV-1 positive
140,160	Europe	OK	OK	IND	FN	FN	FN	FN	FN	425,00	R	5120	R	N	NT	Positive	HIV-1 positive
140,167	Europe	FN	FN	OK	OK	IND	FN	FN	FN	27,10	R	640	R	N	NT	Positive	HIV-1 positive
140,192	Europe	FN	FN	IND	OK	OK	OK	FN	FN	15,00	R	320	R	N	NT	Positive	HIV-1 positive
Initial result not correct in two RDTs																	
140,056	Africa	FN	FN	OK	OK	OK	OK	FN	FN	33,00	R	640	R	N	NT	Positive	Negative
140,111	Africa	FN	FN	OK	OK	OK	OK	FN	FN	57,00	R	640	R	N	NT	Positive	Negative
140,133	Africa	FN	FN	OK	OK	OK	OK	FN	IND	61,40	R	640	R	N	NT	Positive	Negative
140,134	Africa	FN	FN	OK	OK	OK	OK	FN	FN	46,80	R	1280	R	N	NT	Positive	Negative
140,140	Europe	FN	OK	OK	OK	OK	OK	FN	FN	35,40	R	320	R	N	NT	Positive	HIV-1 positive
140,141	Africa	FN	OK	OK	OK	OK	OK	FN	FN	64,80	R	320	R	N	NT	Positive	HIV-1 positive
140,152	Europe	FN	FN	OK	OK	OK	OK	FN	FN	18,20	R	160	R	N	NT	Positive	HIV-1 positive
140,174	Europe	FN	FN	OK	OK	OK	OK	IND	FN	29,30	R	320	R	R	NT	Positive	HIV-1 positive
140,219	Europe	FN	FN	OK	OK	OK	OK	FN	FN	41,50	R	640	R	N	NT	Positive	HIV-1 positive
140,223	Europe	FN	FN	OK	OK	OK	OK	FN	IND	42,50	R	640	R	N	NT	Positive	HIV-1 positive
140,230	South America	FN	FN	OK	OK	OK	OK	FN	FN	53,60	R	320	R	N	NT	Positive	HIV-1 positive
WHO3–0586	Africa	FR	FR	OK	OK	OK	OK	FR	FR	0,02	NR	–	NR	N	Negative	Negative	HIV-1 positive

*ITG* specimens obtained through the ITM clinic; *FR* false reactive, *FN* false non-reactive, *OK* result is concordant with the reference result, *IND* indeterminate result, *S/CO* sample signal/cut-off, *OD/CO* optical density/cut-off, *R* reactive, *NR* non-reactive, *NT* not tested, *N* negative

### Commercial panels

The results obtained for the different panels are summarized in Table 5. Both for the HIV and the TP seroconversion panels, the SD Bioline assay was more sensitive in early detection of antibodies compared with the other three assays and compared to the reference assays.

**Table 5:** Test results of the commercial HIV and TP panels

	SD Bioline HIV/Syphilis Duo	DPP HIV-Syphilis Assay	Multiplo Rapid TP/HIV Antibody Test	Insti Multiplex HIV-1/HIV-2/Syphilis Antibody Test
HIV seroconversion panel	HIV-1/2 antibodies were detected 0.125 specimens earlier compared to the reference assay	HIV-1/2 antibodies were detected 0.25 specimens later compared to the reference assay	HIV-1/2 antibodies were detected 0.125 specimens later compared to the reference assay	HIV-1/2 antibodies were detected at the same time as the reference assay
HIV mixed titre panels	All were correctly detected	88% were correctly detected (22/25; one false reactive, one false non-reactive and one indeterminate)	96% were correctly detected (24/25; one false non-reactive)	All were correctly detected
WHO HIV Reference preparations	All were correctly detected, with the exception of the Group O specimen (indeterminate)	All were correctly detected	All were correctly detected	All were correctly detected
TP seroconversion panels	TP antibodies were detected 2 specimens earlier compared to the reference assay	TP antibodies were detected at the same time compared to the reference assay	TP antibodies were detected 2 specimens later compared to the reference assay	TP antibodies were detected 1 specimen earlier compared to the reference assay
TP mixed titre panels	All were correctly detected	All were correctly detected	All were correctly detected	All were correctly detected
WHO TP Reference preparations	All were correctly detected	All were correctly detected	All were correctly detected	All were correctly detected

## Discussion

Introduction of RDTs in healthcare is extremely important as these tests may result in an accelerated linkage to care and treatment for many people. Advantages of RDTs over traditional laboratory-based in vitro diagnostics are their ability to be used in rural settings with limited laboratory access, their simplicity of execution and the shorter time to result. Dual HIV/Syphilis RDTs have the additional advantage of using the same specimen (serum/plasma or whole blood) and test device for testing the two infections simultaneously. Consequently, syphilis antibody screening can be easily added to the already existing HIV screening programmes without the need for extra blood sampling and extra waiting time for the result. This may be an important step forward in controlling HIV and syphilis infections in vulnerable risk groups and pregnant women. In this respect the WHO published a note in early 2017 in order to provide advice for countries planning to introduce the HIV/syphilis dual test in antenatal care settings.

This study presents data from four HIV/Syphilis RDTs, obtained after evaluation on the same, well characterized, specimen panel by experienced laboratory personnel in a WHO accredited testing laboratory with ISO 15189 and ISO 17025 accreditation. This makes the sensitivity and specificity calculations very comparable between the four assays. Sensitivities and specificities obtained for anti-HIV antibody detection (Table 2) are in line with results from earlier published laboratory evaluations for all four assays [3–7, 9, 11–14, 16–21]. For *Treponema pallidum*, however, the sensitivities obtained in our study were lower compared to earlier published data for all assays, while specificities were comparable. One study, by Fakile et al. [21], reported similar lower sensitivities and specificities for treponemal antibodies.

The sensitivities for *Treponema pallidum* detection of three of the four assays were higher than the minimal clinical sensitivity of 80% as set by the WHO in their product profile, but none of the assays achieved the optimal desired clinical sensitivity of 90%. For all four assays the *Treponema pallidum* specificities were superior to the optimal desired clinical specificity of 95%. Ref http://www.who.int/reproductivehealth/POTC-TPPs-2016.pdf.

The different geographical origins of the study populations, as well as the stage of the syphilis infection at time of sampling, might explain these differences in sensitivities. The natural history of syphilis is complex in that the treponemal and non-treponemal antibody profile varies in the different syphilis stages. Treponemal antibodies (IgG and IgM) appear earlier than the non-treponemal antibodies, they remain detectable for life and do not protect against new infection. On the other hand, non-treponemal antibodies decrease and may disappear over time, especially after successful treatment. For SD Bioline and the Multiplo assay the IFU state that they detect IgG as well as IgM antibodies. Additionally, as per the IFU of INSTI, patients in the early primary stage of infection may test negative due to the test’s lower affinity to IgM antibodies as compared to IgG. The IFU of the DPP assay does not specify the type of antibody but states that individuals with syphilis who are receiving antibacterial therapy may produce false negative results. For our study we made sure that the panel was composed of specimens from different geographic origins but no selection was made based on the syphilis stage.

Laboratory evaluations executed in an ideal situation give a good reflection of the test with optimal performance characteristics. On the other hand, this strength may also be a limitation because it may not reflect the situation in reality, when these RDTs will be performed in the field by health care workers using whole blood finger stick specimens. So far, data obtained from field evaluations are rather scarce. Bristow et al. [8, 11] and Black et al. [13] found sensitivities and specificities for HIV antibodies for SD Bioline HIV/Syphilis Duo ranging from 98.8 to 99.2% and 97.0 to 100%, respectively, and for *Treponema pallidum* from 66.7 to 96.5% and 90.8 to 98.8%, respectively. In field settings, the performance characteristics are lower than those observed in laboratory settings, especially for the TP component (Table 6).

**Table 6:** Test performance characteristics; comparison with earlier studies performed by other groups

	SD Bioline HIV/Syphilis Duo	DPP HIV-Syphilis Assay	Multiplo Rapid TP/HIV Antibody Test	Insti Multiplex HIV-1/HIV-2/Syphilis Antibody Test
Antibodies to HIV				
*Sensitivity (%)*				
This evaluation (95% CI)	100 (98.2–100)	100 (98.2–100)	99.5 (97.2–100)	99.5 (97.2–100)
Previous laboratory evaluations^a^	97.1–100	98.8–100	97.9–100	100
Previous field evaluations^a^	98.8–99.2	NA	93.8	93.8
*Specificity (%)*				
This evaluation	99.5 (97.2–100)	97.5 (94.3–99.2)	99.5 (97.2–100)	93.5 (89.1–96.5)
Previous laboratory evaluations^a^	99.5–100	97.9–98.7	91.9–98.3	95.5
Previous field evaluations^a^	97.0–100	NA	100	100
Antibodies to Treponema pallidum				
*Sensitivity (%)*				
This evaluation (95% CI)	87.0 (81.5–91.3)	86.5 (81.0–90.9)	73.5 (66.8–79.5)	81.0 (74.9–86.2)
Previous laboratory evaluations^a^	72.2–100	82.5–98.8	80.7–94.6	87.4
Previous field evaluations^a^	66.7–96.5	NA	81	81.0
*Specificity (%)*				
This evaluation	99.5 (97.2–100)	100 (98.2–100)	99.5 (97.2–100)	99.0 (96.4–99.9)
Previous laboratory evaluations^a^	96.0–100	96.4–100	88.7–97.2	97.0
Previous field evaluations^a^	90.8–98.8	NA	100	100

SD Bioline previous laboratory evaluations: references [5, 7, 12–14, 16, 17, 20, 21, 23, 24]; SD Bioline previous field evaluations: references [8, 11, 15]; DPP previous laboratory evaluations: references [5, 17–19, 23]; Multiplo previous laboratory evaluations: references [5, 6, 17, 23]; Multiplo previous field evaluations: reference [10]; Insti previous laboratory evaluation: reference [9]

*NA* Not available

^a^range from lowest obtained sensitivity or specificity to highest

For the *Treponema pallidum* positive but HIV negative specimens, 31% were false reactive for HIV antibodies in at least one of the four evaluated assays. The intensity of the HIV test line was much weaker as compared to true positive results. These weakly reactive bands may be a result of non-specific serological cross-reactivity as described in a review by Klarkowski et al., where possible causes of false reactivity on RDTs are discussed [23]. They emphasize that stimulation of immune activation (B-lymphocyte activation), which produces broad-spectrum antibodies, might be a significant cause of cross-reactivity and thus false reactive results. In the same review, no studies were described in which syphilis caused HIV false reactivity. The high rate of HIV false reactivity strengthens the need to ensure the quality of testing and underlines the fact that one reactive test result may not be considered as definitive. Any reactive test should always be confirmed by additional testing, as recommended by WHO [24].

Notably, 7% of the 200 anti-TP positive specimens were false non-reactive for TP antibodies in all four assays, all were TPA and TPPA positive and RPR negative, and another 28 were misclassified by two or three assays. This rather high misclassification rate, by two or more assays, may be the result of some characteristics of the setup of our evaluation. Firstly, the consistency of the specimens. Two of the four evaluated assays (Multiplo and INSTI) are flow through principle RDTs. Flow through (or vertical flow) immunoassays rely on the same basic principles as the more common lateral flow immunoassays (such as DPP and SD Bioline), with the flow of the fluid, vertically versus laterally, as the most obvious difference. Because they are based on vertical flow/absorption of the specimen, the assay’s performance can be influenced by the composition of the specimens, especially when working with stored serum/plasma, as was the case in our evaluation. As specified in the Multiplo’s Instructions For Use (IFU), all specimens were centrifuged and only the clear supernatant was used on the device. Centrifugation was not described in the other IFU and was therefore not performed for INSTI, SD Bioline and DPP. However, approximately 85% of the specimens that were false non-reactive with INSTI (using un-centrifuged specimens) were also misclassified with Multiplo (using clear supernatant), we therefore suggest that the use of centrifuged and clear supernatant did not have an influence on the final results.

Secondly, the number of freeze/thaw cycles that a specimen had already passed through before being used in this evaluation could differ between the RDTs evaluated, and also between initial testing and eventual repeat testing. Castro et al. [25] found that 10 cycles appear to have a minimal effect on the sensitivity of IgG and IgM for serological testing. As all specimens in our panel were stored at − 20 °C in small aliquots, no specimen exceeded more than 2 freeze/thaw cycles, which makes it likely that there was no loss of antibodies.

Another limitation of the current study is the low number of HIV or syphilis non-reactive specimens (200 for each) included in the evaluation. A larger sample size would have given the study results more power. However, we believe that this evaluation reflects the performance of the four assays. A table (Table 7) shows positive and negative predictive values at three different prevalence’s for both markers HIV and Treponema. They are also part of each of the individual reports available for the public on the WHO website.

**Table 7:** Predictive values (%) at different prevalence’s for each of the markers, HIV and *Treponema pallidum*

Prevalence %	SD Bioline HIV/Syphilis Duo		DPP HIV-Syphilis Assay		Multiplo Rapid TP/HIV Antibody Test		Insti Multiplex HIV-1/HIV-2/Syphilis Antibody Test	
	HIV	T pallidum	HIV	T pallidum	HIV	T pallidum	HIV	T pallidum
PPV								
0.1	16.68	14.76	2.44	100	16.61	6.55	0.82	7.29
1	66.89	63.60	20.16	100	66.78	41.42	7.73	44.23
5	91.32	90.10	56.82	100	91.28	78.65	30.40	80.51
NPV								
0.1	100	99.99	100	99.98	100	99.97	100	99.98
1	100	99.86	100	99.85	99.99	99.69	99.99	99.78
5	100	99.20	100	99.22	99.97	98.43	99.97	98.87

*PPV* positive predictive value, *NPV* negative predictive value

Rapid diagnostic tests are mainly developed to be used in the field, by healthcare workers. For that reason it is important that not only the performance characteristics are evaluated but also the operational characteristics. During this study, the operational aspects, such as ease of use, number of steps and ease of interpretation, were assessed by the lab technician who performed the tests (Table 1). The Multiplo and INSTI assay are both vertical flow immunoassays and therefore need more steps to be performed before the result can be interpreted: the technician must be very alert. In our experience, in some cases the test bands/dots were hardly visible, making interpretation difficult and therefore resulting in false reactive, false non-reactive or indeterminate results. Proper training is essential, not only for the vertical flow but also for the lateral flow RDTs, especially when working with non-lab personnel.

## Conclusion

In conclusion, the results of this laboratory evaluation suggest that combined HIV/Syphilis rapid diagnostic tests are a useful tool for the detection of both infections using the same device, and can increase the accessibility of HIV/Syphilis diagnosis for difficult to reach populations in the world. However, further evaluations should be conducted to assess the feasibility and acceptability of such assays among healthcare workers in the field. The tests under investigation could further improve in accuracy, especially in the detection of treponemal antibodies, but are promising enough to be introduced into screening programmes.

## Abbreviations

EIA: Enzyme immunoassay
IFU: Instructions for use
ITM: Institute of Tropical Medicine
RDT: Rapid diagnostic test/rapid point-of-care test
STI: Sexually transmitted infection
TP: Treponemal
TPPA: *Treponema pallidum* passive particle agglutination
WHO: World Health Organisation
